# Protective effect of α-lipoic acid on islet cells co-cultured with 3T3L1 adipocytes

**DOI:** 10.3892/etm.2012.601

**Published:** 2012-06-07

**Authors:** YUFAN WANG, WEIPING DONG, XIAOYING DING, FENG WANG, YUFEI WANG, XINYA CHEN, LONG YU, XIAOHUA LI, AIFANG ZHANG, YONGDE PENG

**Affiliations:** 1 Department of Endocrinology and Metabolism;; 2 Diabetes Research Laboratory;; 3 Central Experimental Laboratory, Shanghai Jiao Tong University Affiliated First People’s Hospital, Shanghai 200080;; 4 State Key Laboratory of Genetic Engineering, Institute of Genetics, Fudan University, Shanghai 200433, P.R. China

**Keywords:** α-lipoic acid, 3T3L1 adipocytes, islet cells, oxidative stress, co-culture

## Abstract

Obesity and β-cell dysfunction due to oxidative stress impact the pathogenesis of type 2 diabetes mellitus. We co-cultured 3T3L1 adipocytes and islet cells in the presence or absence of the antioxidant α-lipoic acid (LA) and assayed the effects of the adipocytes and LA on the secretion of insulin by the islet cells and on the activities of factors involved in secretion and oxidative stress. At low glucose concentrations (2.8 mmol/l), the presence of adipocytes (co-culture) increased insulin secretion compared with islet cells cultured alone (control) and this increase was diminished by LA (co-culture plus LA). At high glucose concentrations (22 mmol/l), insulin secretion levels were similar for all islet groups, resulting in a restoration of the stimulation index in the presence of LA. The mRNA levels of the glucose-stimulated insulin secretion (GSIS) genes glucokinase, glucose transporter 2 and Kir6.2 were downregulated under co-culture and co-culture plus LA conditions. Protein and tyrosine phosphorylation levels of insulin receptor-β and insulin receptor substrate-1 were decreased under co-culture conditions and were restored by LA treatment. Cellular malondialdehyde levels increased in the co-cultured islets and this increase was blocked by LA. The mRNA levels of superoxide dismutase and catalase were reduced under co-culture conditions and these reductions were eliminated by the addition of LA. In conclusion, 3T3L1 adipocytes disturb insulin secretion and induce islet dysfunction. The effects may be mediated by multiple pathways, which include downregulation of GSIS gene expression, suppression of islet cell insulin signaling and the induction of oxidative stress. LA may protect islet cells via activation of islet cell insulin signaling and the mRNA expression of antioxidant enzymes.

## Introduction

Recent advances in obesity research have led to the recognition that adipose tissue is an active endocrine organ that secretes multiple bioactive factors, termed adipokines (1). Adipokines are involved in diverse biological functions, including energy homeostasis, insulin sensitivity, lipid metabolism, inflammation and immunity (2). Accompanying insulin resistance, β-cell dysfunction is another significant pathophysiological change associated with type 2 diabetes mellitus (T2DM). Following the discovery of receptors for several adipokines, including leptin (3), tumor necrosis factor-α (4) and interleukin-6 (5) in pancreatic β cells, the possible involvement of these factors in β-cell dysfunction has been proposed. Although not extensively investigated, several observations indicate that adipokines influence β-cell function and play a role in the development of β-cell dysfunction in T2DM (6).

Diabetes mellitus is strongly associated with oxidative stress, which may be a consequence of either the increased production of free radicals or reduced antioxidant defenses. α-lipoic acid (LA) is a potent antioxidant which scavenges free radicals and recycles other antioxidants to reduce oxidative stress. As a result, it is widely used in the treatment of diabetic neuropathy and has the potential for treating several aspects of diabetes pathology since it prevents β-cell destruction (a cause of T1DM) (7) and enhances glucose uptake in T2DM (8). Recently, R-LA (dextrogire) was found to have protective effects on MIN6 and isolated rat islets chronically exposed to oleic acid (9). These observations prompted us to explore the detailed mechanism by which LA acts on islet cell dysfunction.

To investigate the integrated effects of adipocytes on rat islet cells and the potential of LA to mediate a protective effect, we established a co-culture system comprising differentiated 3T3L1 adipocytes and islet cells. The effects of the adipocytes and LA on islet cell function were monitored by assessing the changes in insulin secretion and gene expression and the protein levels of factors involved in insulin secretion, signaling and oxidative stress under co-culture conditions and in the presence or absence of LA.

## Materials and methods

### Reagents

3T3L1 cells were provided by Dr Tang Qiqun (Shanghai Medical College of Fudan University, Shanghai, China). Dulbecco’s modified Eagle’s medium (DMEM), penicillin/streptomycin and fetal bovine serum (FBS) were purchased from Invitrogen (Carlsbad, CA, USA). Dexamethasone, isobutylmethylxanthine, LA and protein A/G were purchased from Sigma-Aldrich (St. Louis, MO, USA). Insulin was purchased from Eli Lilly (Shuzhou, China). Antibodies were obtained as follows: insulin receptor-β (IR-β) and insulin receptor substrate-1 (IRS-1) antibodies (Cell Signaling Technology, Danvers, MA, USA); and anti-phospho-tyrosine antibody, clone 4G10 (Upstate Biotechnology, Inc., Lake Placid, NY, USA). Membrane inserts for 6-well culture dishes with a pore size of 0.4 *μ*m and insert companion plates were supplied by Corning (New York, NY, USA). Collagenase P was purchased from Roche Diagnostics GmbH (Mannheim, Germany). Ficoll 400 was purchased from GE Healthcare (Uppsala, Sweden).

### Animals

Sprague-Dawley rats (10–12 weeks old, 300–350 g) were purchased from the Chinese Academy of Sciences (Shanghai, China). They were given free access to tap water and standard pelleted chow following the regulations of the State Science and Technology Commission for the care and use of laboratory animals.

### 3T3L1 cell culture and differentiation

3T3L1 pre-adipocytes were grown to confluence in an incubator equilibrated with 5% CO_2_ at 37°C on membrane inserts in DMEM containing 10% FBS. At 2 days post-confluence (day 0), differentiation was induced by the addition of isobutylmethylxanthine (0.5 mmol/l), dexamethasone (1 *μ*mol/l) and insulin (0.17 *μ*mol/l) in DMEM containing 25 mmol/l glucose and 10% FBS. After 2 days, the isobutylmethylxanthine and dexamethasone were removed and the cells were incubated in DMEM with insulin for an additional 2 days. On day 4, the medium was switched back to DMEM (without insulin supplementation) plus 10% FBS and replenished every 2 days (10). The cells were ready for use after 10 days of differentiation.

### Rat islet isolation and purification

The isolation method was a modification of the method of Sakata *et al* (11). Pancreatic islets were isolated from anesthetized Sprague-Dawley rats following distention of the pancreas by perfusion via the common bile duct with collagenase P solution (1.5 mg/l). The distended pancreas was removed and incubated at 37°C for 14 min to promote collagenase digestion. The incubated mixture was filtered through a 600-*μ*m nylon screen and the filtrate was washed by performing 3 cycles of centrifugation and resuspension in Hanks’ solution. Finally, the islets were purified by Ficoll-mediated discontinuous gradient centrifugation. The islets were further tested for purity by dithizone (DTZ) staining (12) and for viability with acridine orange and propidium iodide (13). The purified islets were maintained in DMEM (containing 5.6 mmol/l glucose and 10% FBS) for 2–3 h before co-culturing with adipocytes. For each individual experiment, islet cells were seeded in 6-well culture dishes at a density of approximately 200 islets per well.

### Co-culture of 3T3L1 adipocytes and islet cells

After differentiating *in vitro* on membrane inserts for 10 days, the 3T3L1 adipocytes were transferred to culture plates containing purified islets. The two cell types shared the same culture medium (DMEM containing 5.6 mmol/l glucose and 10% FBS), but were separated by the membrane inserts. Co-culturing was conducted for 48 h. The integrity of the cells was routinely checked at the end of the co-culture period by light microscopy. There were three groups: a control group (islets alone), a co-culture group and a co-culture plus LA group. For the latter group, the islets were exposed to 0.4 mmol/l LA during the co-culture period.

### Insulin secretion and insulin content

After co-culturing for 48 h, the adipocytes were removed and the islets were washed twice with Krebs-Ringer-HEPES buffer (KRBH: 115 mmol/l NaCl, 5.4 mmol/l KCl, 2.38 mmol/l CaCl_2_, 0.8 mmol/l MgSO_4_, 1 mmol/l Na_2_HPO_4_, 10 mmol/l HEPES, 0.5% BSA, pH 7.35) containing 2.8 mmol/l glucose. Eight purified islets of approximately the same size were selected and preincubated for 1 h in KRBH containing 2.8 mmol/l glucose. They were subsequently incubated for 1 h in KRBH containing either 2.8 or 22 mmol/l glucose (14). The supernatant was collected and assayed for insulin secretion using a rat insulin radioimmunoassay kit (Linco Research, St. Charles, MO, USA) according to the manufacturer’s instructions. The total cellular insulin content was extracted using 75% ethanol containing 1.5% (vol/vol) HCl for 24 h at 4°C (15) and was normalized by cellular protein content.

### RNA extraction and real-time RT-PCR

RNA was isolated from islets using TRIzol (Invitrogen). cDNA was synthesized from 1 *μ*g total RNA using AMV Reverse Transcriptase (Promega, Madison, WI, USA) in a 20-*μ*l reaction volume. The PCR products were quantified fluorometrically using SYBR^®^ Premix Ex Taq™ (2X) (Takara Bio, Inc., Shiga, Japan) according to the manufacturer’s instructions. The cycling parameters were 95°C for 2 min, then 45 cycles of 94°C for 2 sec and 60°C for 30 sec. The primer sequences used are presented in Table I. GAPDH expression in each sample was used as a control. All reactions were performed in triplicate and the data were normalized to values of GAPDH, and evaluated using the 2^−ΔΔCT^ method. Expression levels are presented as the fold changes of the transcripts of the respective genes relative to the controls.

### Immunoprecipitation and western blotting

The cell lysates were extracted with lysis buffer (Cell Signaling Technology), 1% PMSF and a complete protease inhibitor cocktail. Lysis was carried out with gentle rotation at 4°C for 20 min. The lysate was then centrifuged at 12,000 × g for 5 min at 4°C to remove the insoluble materials. The protein concentrations were determined using the BCA Protein Assay kit (Novagen, Madison, WI, USA). For immunoprecipitation, the supernatant was incubated with Protein A or G agarose beads for 1 h at 4°C for pre-cleaning. Subsequently the pre-cleaned supernate was incubated with Protein A or G agarose beads carrying a pre-absorbed antibody (IR-β or IRS-1, diluted 1:50) for 5 h at 4°C with gentle rotation. The resulting immunopellet was collected by centrifugation and washed 5 times with the lysis buffer. The cell lysates or immunoprecipitates were boiled with 2X loading buffer for 8 min and centrifuged, and the complete supernatant was used for SDS-PAGE analysis. The proteins were transferred from gel to nitrocellulose sheets and blocked with 5% fat-free milk. The blots were probed with various primary antibodies (IR-β, IRS-1 and 4G-10, diluted 1:500, β-actin diluted 1:2000). The proteins were detected by enhanced chemiluminescence using horseradish peroxidase-labeled secondary antibodies (diluted 1:5000, Millipore, Billerica, MA, USA).

### Assay of malondialdehyde (MDA) levels

MDA concentrations were determined by spectrophotometric assays according to the manufacturer’s instructions (Nanjing Jianchen Tech, Nanjing, China).

### Statistical analysis

Data are expressed as the mean ± SE. Statistical analysis was performed by one-way ANOVA followed by the least-significant difference test or Tamhane’s T2 test. P<0.05 was considered to indicate a statistically significant result.

## Results

### Effect of LA on insulin secretion by rat islets co-cultured with 3T3L1 adipocytes

**Figure 1:**
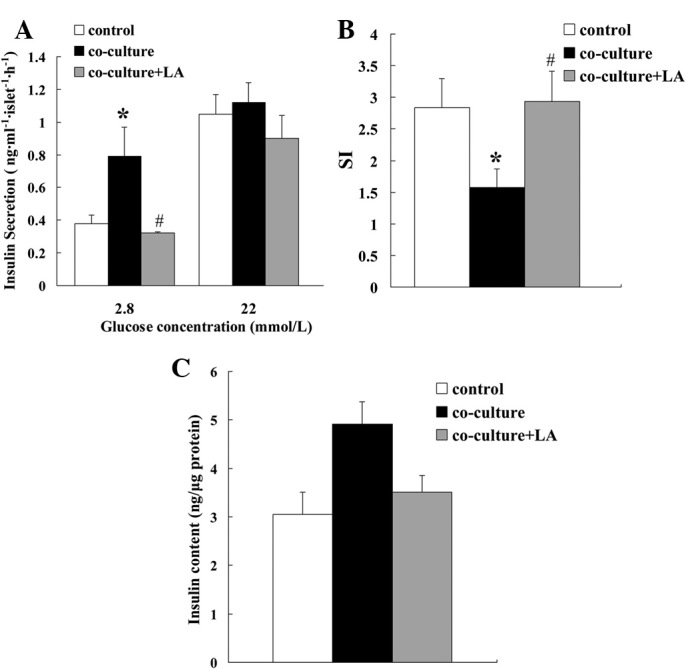
LA restored the SI of rat islets co-cultured with 3T3L1 adipocytes. Islet cells were cultured in the absence (control) or presence of adipocytes (co-culture) and in the presence of adipocytes + LA (co-culture + LA) for 2 days, after which the adipocytes were removed and the islets were incubated in KRBH with 2.8 mmol/l (low glucose) or 22 mmol/l (high glucose) glucose. (A) The insulin secretion of the three groups (control, co-culture and co-culture islets + 0.4 mmol/l LA) at low and high glucose concentrations. (B) SI of the three islet groups. (C) The total cellular insulin contents of the three islet groups were determined. Data are the mean ± SE from four independent experiments. Values are statistically significant (^*^P<0.05 compared with the control group, ^#^P<0.05 compared with the co-culture group). LA, α-lipoic acid; SI, stimulation index.

To examine the effects of adipocytes and LA on islet cell function, we first evaluated insulin secretion levels. The islets were isolated and incubated in KRBH containing either 2.8 or 22 mmol/l glucose. At the lower glucose level, insulin secretion levels were higher for the islets that had been co-cultured with adipocytes (co-culture) than those that had not (control). The presence of LA (co-culture plus LA) eliminated this increase in secretion. At the higher glucose level, the insulin secretion levels were similar for the three differently treated islet groups (Fig. 1A). The stimulation index (SI, insulin release at high glucose/low glucose) of the co-cultured islets was lower than that of the control islets, but was restored by the addition of LA (Fig. 1B). The cellular insulin content was similar for the three groups (Fig. 1C).

### Effect of LA on glucose transporter 2 (GLUT2), glucokinase (GCK) and Kir6.2 mRNA levels in rat islets co-cultured with 3T3L1 adipocytes

**Figure 2:**
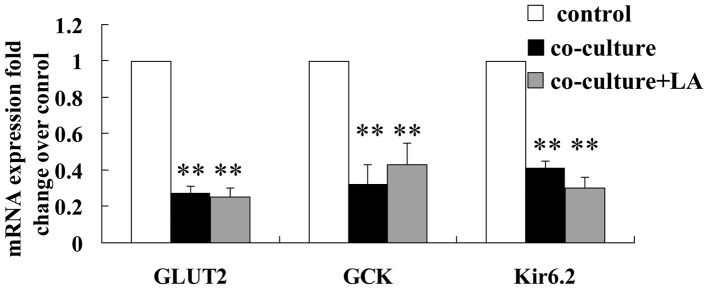
Effects of LA on GSIS gene expression in rat islets co-cultured with 3T3L1 adipocytes. After co-culturing, islets from the three treatment groups were selected and the mRNA expression levels of GCK, GLUT2 and Kir6.2 were measured by real-time RT-PCR. The data were normalized to glyceraldehyde 3-phosphate dehydrogenase. The fold changes in expression were calculated relative to the respective control group. All data are the mean ± SE from 3–5 independent experiments. Values are statistically significant (^**^P<0.01 compared with the control group). LA, α-lipoic acid; GSIS, glucose-stimulated insulin secretion; GCK, glucokinase; GLUT2, glucose transporter 2.

Glucose enters the β cells through GLUT2. Elevated concentrations of glucose within the β cells enhance glucose metabolism resulting in an increased cellular adenosine triphosphate/adenosine diphosphate (ATP/ADP) ratio and subsequently leads to the closure of KATP channels, membrane depolarization, an influx of extracellular calcium and exocytosis of insulin granules. GLUT2, GCK and Kir6.2 are the key genes involved in the process of glucose-stimulated insulin secretion (GSIS). The mRNA levels of these factors were examined in the three groups of islets following 48 h of culturing to assess whether the presence of adipocytes and LA altered their expression. The expression levels in the co-cultured islets were lower than in the control islets (downregulated to 27, 32 and 40%, respectively). Meanwhile, the mRNA levels of these three factors were not altered by the addition of LA (Fig. 2).

### Effect of LA on protein expression and tyrosine phosphorylation of IR-β and IRS-1 in rat islets co-cultured with 3T3L1 adipocytes

**Figure 3:**
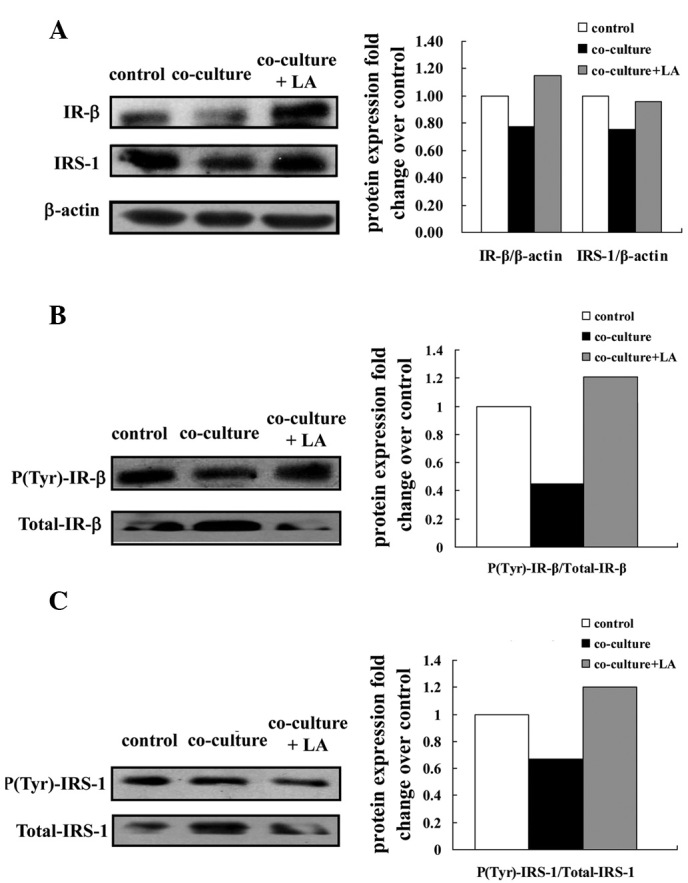
LA restored protein expression and tyrosine phosphorylation of IR-β and IRS-1 in rat islets co-cultured with 3T3L1 adipocytes. Rat islets were co-cultured with 3T3L1 adipocytes for 48 h. Fat cells were then removed and the islets were collected by cell lysis and immunoprecipitation of IR-β or IRS-1. (A) The immunoprecipitates were resolved by SDS-PAGE and immunoblotted with anti-IR-β or anti-IRS-1 antibodies. (B and C) The immunoprecipitates were resolved by SDS-PAGE and immunoblotted with 4G-10 antibody to detect the tyrosine phosphorylated proteins P(Tyr) (upper panels). Signals were visualized using enhanced chemiluminescence detection. The fold changes were calculated relative to the respective control group. The immunoblots are representative of ≥2 independent experiments. LA, α-lipoic acid; IR-β, insulin receptor-β; IRS-1, insulin receptor substrate-1.

Insulin has an significant autocrine action on β cells, which is necessary for maintaining normal secretion. We investigated certain factors that participate in the insulin-signaling pathway. As depicted in Fig. 3, the protein levels of IR-β and IRS-1 were altered by the presence of the adipocytes. The protein levels of IR-β and IRS-1 in the co-cultured islets decreased to ∼77 and 75%, respectively, of their values in the control islets (Fig. 3A). The tyrosine phosphorylation levels of these proteins were reduced to ∼45 and 67%, respectively, of their values in the control islets (Fig. 3B and C). The addition of LA upregulated the IR-β and IRS-1 protein expression levels 1.49- and 1.28-fold, respectively, and increased their tyrosine phosphorylation levels 2.68-and 1.79-fold, respectively, compared with the co-cultured islets (Fig. 3).

### Effect of LA on oxidative stress biomarkers in rat islets co-cultured with 3T3L1 adipocytes

**Figure 4:**
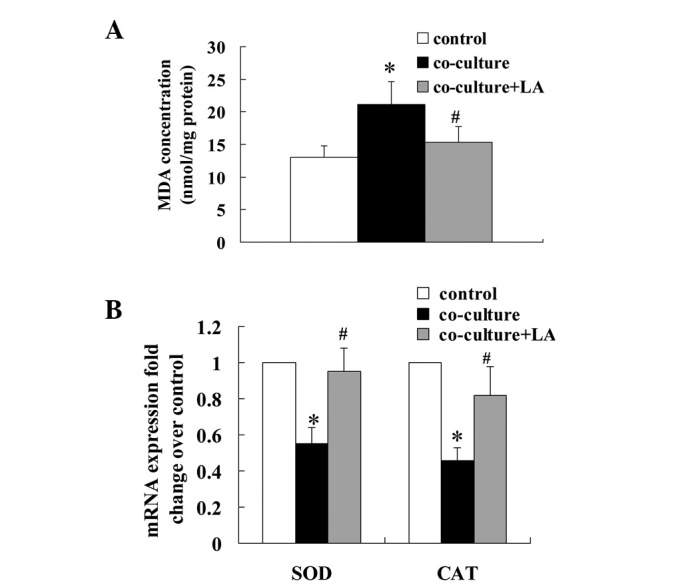
LA restored MDA levels and the mRNA levels of SOD and CAT in rat islets co-cultured with 3T3L1 adipocytes. Rat islets were incubated for 48 h in co-culture with 3T3L1 adipocytes. Adipocytes were then removed, and the islets were collected by cell lysis. (A) MDA concentration of the three islet treatment groups. (B) SOD and CAT mRNA expression levels in the three islet treatment groups measured by real-time RT-PCR. All data are the mean ± SE from 4–5 independent experiments. Values are statistically significant (^*^P<0.05 compared with the control group, ^#^P<0.05 when compared with the co-cultured group). LA, α-lipoic acid; MDA, malondialdehyde; SOD, superoxide dismutase; CAT, catalase.

Since LA is a potent antioxidant, we examined the cellular content of the lipid peroxidation factor MDA and the mRNA levels of the antioxidant enzymes superoxide dismutase (SOD) and catalase (CAT). MDA levels were increased in the co-cultured islets compared with the control islets (21.08±12.36 vs. 13.03±5.98 nmol/mg; Fig. 4A). This increase was inhibited by the addition of LA. The mRNA levels of SOD and CAT in the co-cultured islets were significantly decreased to 55 and 46%, respectively, of their levels in the control islets and this reduction was blocked by treatment with LA (Fig. 4B).

## Discussion

Adipocytes produce adipokines, which travel to distant sites (including the pancreas) where they may exert deleterious effects. To analyze the metabolic effects of adipokines, most studies have investigated the effects of recombinant proteins *in vitro*. However, this approach may not reflect what is happening *in vivo*, for example the extensive crosstalk between bioactive factors (16). The co-culture system (differentiated 3T3L1 adipocytes and islet cells) characterized in this study may be a new and potentially more physiologically relevant tool for investigating the effects of adipokines on rat islet dysfunction.

In this study, we found that at a low glucose concentration (2.8 mmol/l), co-culturing the islets with adipocytes resulted in increased insulin secretion levels. However, at high glucose levels (22 mmol/l), the co-cultured and control islets secreted similar levels of insulin resulting in a decreased SI. Zhao *et al* have previously established a co-culture system of differentiated 3T3L1 adipocytes and MIN6 cells (17). In contrast to our findings, they found that after seven days of co-culturing, insulin secretion by the co-cultured MIN6 cells decreased at low glucose levels, and tolbutamide (a KATP blocker) stimulated a significant increase in insulin secretion levels in the control but not the co-cultured MIN6 cells (17). The difference between the findings of the two studies may result from a difference in culture time (2 days in the current study compared with 7 days for the earlier study) and/or in cells (we used isolated rat islets while the earlier study used MIN6 cells). Tolbutamide was used in the earlier study since the MIN6 cells did not respond normally to high glucose levels. In support of our findings, the previous study revealed that pancreatic islets exposed to free fatty acid (FFA) for 48 h exhibited enhanced basal insulin secretion and an impaired response of the β cells to glucose stimulation (18). Taken together, it is expected that short-term exposure (2 days) to adipocytes may overactivate the islet cells, resulting in an augmentation of basal insulin release and a reduced susceptibility to high glucose levels, whereas longer exposure (7 days) to adipocytes may result in exhaustion of the insulin secretion by the β cells. Moreover, the findings in these studies are consistent with the pathophysiological process of hyperinsulinemia at the early stage of T2DM and the progressive impairment of insulin secretion over time.

In agreement with the findings of Zhao *et al*, we found that the cellular insulin contents were comparable for the co-cultured and control islets. These findings suggest that the adipokines do not increase insulin secretion by enhancing insulin synthesis. The decreases in the mRNA levels of GLUT2, GCK and Kir 6.2 suggest that adipokines downregulate the mRNA levels of GSIS factors prior to a drop in insulin secretion levels.

Insulin-producing pancreatic β cells are targets for insulin action. Insulin affects a variety of cellular processes, including transcription, translation, glucose and lipid metabolism, ion flux, cell proliferation, cell size and β-cell apoptosis (19). Insulin resistance, in addition to peripheral targets including liver, muscle, fat and brain, may also affect β cells. In our study, the protein and tyrosine phosphorylation levels of IR-β and IRS-1 decreased in the co-cultured islets, which suggests that the adipocytes downregulate the ability of insulin to signal to rat β cells. An increasing body of evidence suggests that β-cell insulin resistance is coupled with β-cell dysfunction and apoptosis (20). Furthermore, β-cell insulin resistance may add to the deterioration of β-cell function and therefore accelerate the progression of T2DM (19).

The beneficial effects of LA on the co-cultured islets may result from its ability to act as a direct mitochondrial antioxidant, phase 2 antioxidant enzyme inducer, energy enhancer or enzyme cofactor (21). In our study, the addition of LA to the co-culture reduced insulin secretion at low glucose levels whereas at high glucose levels it had no significant affect on insulin secretion. These findings indicate that LA suppresses the adipocyte-induced increase in insulin release when glucose levels are low and promotes insulin secretion when they are high.

In a previous study, it was found that the administration of LA to obese rats resulted in increased insulin-stimulated glucose disposal in the whole body and in skeletal muscle (22). LA has also been reported to directly activate tyrosine kinase in 3T3L1 cells (23). The upregulated expression of factors involved in the insulin signaling pathway following LA supplementation in our study indicates that LA reinforces the insulin sensitivity of islet cells in addition to that of peripheral insulin targets. Since the insulin-signaling pathway may affect β-cell insulin release, its activation may contribute to the restored islet secretion of the co-cultured islets treated with LA.

MDA is one of the most frequently used indicators of lipid peroxidation. SOD converts intracellular superoxide radicals into hydrogen peroxide which is decomposed by CAT to form water. SOD and CAT are major antioxidant enzymes. A number of studies suggest that excessive concentrations of reactive oxygen species (ROS) cause pancreatic islet β-cell dysfunction and impair the action of insulin. A recent study revealed that oxidative stress was involved in a FFA-induced decrease in β-cell secretory function and that antioxidants prevented the loss of secretory function (14). Pancreatic β cells have low antioxidant defenses and are thus susceptible to ROS-induced decreases in function and viability (24). As shown in Fig. 4, MDA cellular levels were increased in the co-cultured islets and this increase was inhibited by LA. This suggests that the adipocytes increased the peroxidation of lipids and that the antioxidative effect of LA blocked this effect. The mRNA expression levels of SOD and CAT were decreased in the co-cultured islets, which suggests that the adipocytes inhibit this antioxidative pathway. LA restored the levels of SOD and CAT, possibly reflecting an increase in antioxidant activity. Oxidative stress, the prevalence of oxidant factors over antioxidant mechanisms, plays a central role in the pathogenesis and progression of diabetes and its complications. Hence, it is possible that a substance known to reduce oxidative stress *in vivo* may reduce the progression of cell damage in clinical diabetes (25). Our findings suggest that LA reduces oxidative stress in islet cells by alleviating lipid peroxidation or by increasing the mRNA levels of antioxidant enzymes which detoxify free radicals.

In conclusion, the co-culture system of 3T3L1 adipocytes and islet cells may be a new model for investigating islet lipotoxicity. The presence of 3T3L1 adipocytes disturbs insulin secretion by the islet cells and may induce islet cell dysfunction. The effects may be mediated by multiple pathways, including the downregulation of GSIS gene expression, the suppression of islet cell insulin signaling and the induction of oxidative stress. LA, an antioxidant, may protect islet cells by the activation of insulin signaling in islets and the upregulation of the mRNA expression levels of antioxidant enzymes. Our findings raise the possibility that supplementation with LA may be an effective strategy for preventing β cells from lipotoxicity.

## Figures and Tables

**Table I:** Primer sequences used in real-time RT-PCR amplification of cDNA prepared from rat islet cells of three different groups.

Rat genes	Forward primer (5′→3′)	Reverse primer (5′→3′)
GLUT2	CTCATAGTCACACCAGCACATACG	CAAGCCACCCACCAAAGAACG
GCK	CCTGGGCTTCACCTTCTCCTTC	CCTCACATTGGCGGTCTTCATAG
Kir6.2	CCTGGCCATCCTTATTCTGA	CTTTTTCGGAGGTCCCCTAC
SOD	CTTGGGCAAAGGTGGAAATGAAG	ACAGTTTAGCAGGACAGCAGATG
CAT	AGATACCTGTGAACTGTCCCTACC	CGTATAGAATGTCCGCACCTGAG
GAPDH	ACTCCCATTCTTCCACCTTTGATG	TCCACCACCCTGTTGCTGTAG

GLUT2, glucose transporter 2; GCK, glucokinase; SOD, superoxide dismutase; CAT, catalase.

## Abbreviations:

LA: α-lipoic acid;
IR-β: insulin receptor-β;
IRS-1: insulin receptor substrate-1;
SOD: superoxide dismutase;
CAT: catalase;
T2DM: type 2 diabetes mellitus;
DMEM: Dulbecco’s modified Eagle’s medium;
FBS: fetal bovine serum;
KRBH: Krebs-Ringer-HEPES buffer;
MDA: malondialdehyde;
GLUT2: glucose transporter 2;
GCK: glucokinase;
GSIS: glucose-stimulated insulin secretion;
FFA: free fatty acid;
ROS: reactive oxygen species
